# Can Acupuncture Be Effective in Treating Insufficiently Dilated Cervix Following Intrapartum Uterine Torsion in Cows?

**DOI:** 10.1002/vms3.71080

**Published:** 2026-07-06

**Authors:** Eva Maria Erteld, Sait Sendag, Mehmet Yildiz, Axel Wehrend

**Affiliations:** ^1^ Veterinary Clinic for Reproductive Medicine and Neonatology Justus‐Liebig‐University Giessen Germany; ^2^ Veterinary Clinic for Obstetrics and Gynecology Van Yuzuncu Yil University Van Türkiye

**Keywords:** acupuncture, cow, uterine torsion

## Abstract

**Background:**

This prospective clinical field study evaluated the effect of acupuncture on cervical dilatation following manual correction of intrapartum uterine torsion in cows.

**Methods:**

A total of 93 cows of different breeds and ages diagnosed with intrapartum uterine torsion were randomly assigned to two groups: Group I (*n* = 45), which received standardised acupuncture prior to retorsion, and Group II (*n* = 48), which served as a control without acupuncture treatment. Acupuncture was applied approximately 15 min before the initiation of the retorsion procedure. During this interval, all animals underwent a standardised clinical assessment of the birth canal, including manual evaluation of cervical passability based on predefined reference dimensions of the examiner's hand and tissue elasticity. This ensured consistent baseline characterisation of cervical status prior to retorsion. The acupuncture needles were inserted intramuscularly at predefined anatomical points and remained in situ throughout the subsequent obstetrical management, including the retorsion procedure and, where applicable, until completion of fetal delivery.

**Results:**

There was no significant difference in the degree of cervical dilatation immediately after retorsion between the two groups (*p* = 0.60). However, the interval between successful retorsion and calf delivery was significantly shorter in the acupuncture group compared with the control group (12.2 ± 1.2 min vs. 33.0 ± 1.3 min; *p* = 0.002). Surgical intervention for delivery was required less frequently in cows treated with acupuncture, although this difference did not reach statistical significance.

**Conclusions:**

Acupuncture may represent a supportive therapeutic option in bovine obstetrics following uterine torsion. The observed reduction in time to delivery suggests potential clinical relevance, particularly in cases complicated by insufficient cervical dilatation. These findings support the consideration of acupuncture as an adjunctive, field‐applicable strategy in bovine obstetrical practice, although further controlled studies are required to clarify the underlying physiological mechanisms.

## Graphical abstract

This study evaluates the clinical efficacy of acupuncture as an adjunct therapy in the management of intrapartum bovine uterine torsion. In a randomised field trial involving 93 cows, the effects of acupuncture on cervical status and delivery outcomes were assessed. While the degree of cervical dilatation immediately following manual retorsion did not show a significant difference between the acupuncture and control groups, the interval from successful retorsion to fetal delivery was significantly reduced in the acupuncture‐treated animals (*p* = 0.002). These findings suggest that acupuncture may support functional cervical dilatation and improve obstetrical efficiency, representing a practical and beneficial supportive strategy in bovine clinical practice

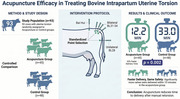

## 1 Introduction

Torsio uteri intrapartum is a common obstetric disorder in cattle, accounting for 5 to 31.8% of dystocia cases. It interrupts the natural birth process and usually requires veterinary obstetric care (Mock et al. 2015; Ulrich and Wehrend 2025). Despite successful conservative retorsion, vaginal delivery often cannot be achieved, particularly when mechanical dilatation of the cervix is not successful. In cattle, this situation often requires a caesarean section (Wehrend and Bostedt 2003; Wehrend and Bostedt 2005). However, in some cases, due to impaired venous circulation associated with both the degree and duration of torsion, surgical procedures on the uterus also involve significant risks due to reduced suture‐holding capacity (Klein and Wehrend 2006; Sendag et al. 2023).

In this context, a potential effect on the cervical opening after retorsion could be economically important (Sickinger et al. 2020; Sendag et al. 2024). Acupuncture is defined as the targeted therapeutic modulation of physiological functions through needle stimulation at specific points on the body surface (Steiss 2003). Furthermore, acupuncture needles have been formally recognised as medical devices by the US Food and Drug Administration (FDA), reflecting their established role in clinical practice (Vickers 1997). The underlying principle is based on the assumption that stimulation of these defined acupuncture points induces reflex‐mediated responses that influence the function of corresponding internal organs. These effects are thought to be mediated via neurophysiological pathways, including activation of somatic afferent fibres, modulation of autonomic nervous system activity, and regulation of regional blood flow. In this context, acupuncture may contribute to the restoration of physiological homeostasis and support endogenous regulatory mechanisms, rather than acting as a direct pharmacological intervention (Egerbacher and Layroutz 1996). In addition, acupuncture has been reported to exert its effects through the stimulation of endogenous opioids and modulation of neurotransmitters such as serotonin and dopamine, leading to analgesic and sedative effects as well as improvement in motor function. These neurochemical mechanisms may further support labour progression by reducing stress and facilitating coordinated uterine activity (Cayir et al. 2014). Evidence from human obstetrics indicates that acupuncture may facilitate cervical dilatation and support labour progression (Hu et al. 2025). These findings suggest that acupuncture could represent a complementary therapeutic approach in the management of parturition‐related disorders. In veterinary medicine, early reports have described a potential positive influence of acupuncture on inadequate cervical opening in cattle (Kothbauer and Zerobin 1977; Westermayer 1979). More recent experimental data further suggest that acupuncture may contribute to placental expulsion in cows by preventing a reduction in metalloproteinase type 2 (MMP2) activity within caruncular tissue (Hiebel et al. 2019).

Despite these indications, the effect of acupuncture on cervical dilatation following uterine retorsion in cattle has not yet been systematically investigated under clinical conditions. Therefore, the present study aimed to evaluate the efficacy of a standardised acupuncture protocol on cervical dilatation after uterine retorsion in dairy cows under field conditions and in a large clinical cohort.

## 2 Material and Methods

### 2.1 Ethical Approval

This study was conducted under routine field conditions in dairy cows diagnosed with intrapartum uterine torsion. All diagnostic and therapeutic procedures, including acupuncture, were performed as part of standard veterinary obstetrical management and did not involve any additional experimental manipulation or invasive intervention beyond established clinical practice. As no procedures were undertaken exclusively for research purposes and no additional risk, distress, or harm was imposed on the animals, formal approval from an institutional animal ethics committee was not considered mandatory under the applicable veterinary regulatory framework. Nevertheless, ethical approval for the study protocol was obtained from the Ethics Committee of  Justus Liebig University Giessen (Animal Welfare Officer‐Ethics review: 1 December 2025). Informed consent was obtained from all animal owners prior to inclusion in the study.

### 2.2 Animals

During a 1.5‐year observation period, cases of intrapartum uterine torsion in cattle from several dairy farms in Justus Liebig University Giessen, were prospectively enrolled in the study. A total of 93 cows of different breeds (German Brown Swiss, German Holstein, and German Simmental) and varying ages, diagnosed with intrapartum uterine torsion, were included in the study. The study population comprised both primiparous and multiparous animals. In the majority of cases, uterine torsion was initially suspected by the owner based on abnormal labour progression. The definitive diagnosis was established by vaginal and rectal examination performed by the same experienced veterinary surgeon in all cases to ensure diagnostic consistency. The following clinical parameters were assessed during rectal and vaginal examination: the direction of torsion (clockwise or counterclockwise), determined by the orientation of spiral folds within the birth canal and the tension of the contralateral broad ligament (ligamentum latum uteri); the degree of torsion, recorded in 90° increments based on fetal position, the extent of vaginal fold formation, and rectal palpation of the torsion site; the localisation of the torsion (cranial or caudal to the cervix), identified according to the position of the spiral folds; and fetal viability.

All cows diagnosed with intrapartum uterine torsion (*n* = 93) were randomly allocated to treatment groups using sealed envelopes prepared in advance by the study supervisor. The envelopes were opened on‐site in the stall immediately prior to intervention. Inclusion criteria were intrapartum uterine torsion and feasibility of manual retorsion. Exclusion criteria comprised antepartum uterine torsion, pathological premature parturition, and severe torsion exceeding 360°. The animals were randomly assigned to two study groups. Group I (*n* = 45) received standardised acupuncture treatment prior to manual retorsion of the uterus. Group II (*n* = 48) served as the control group and underwent retorsion without acupuncture, receiving standard obstetrical management only. All examinations were performed by the same experienced veterinarian using predefined and standardised criteria to minimise variability.

### 2.3 Acupuncture Point Selection

**FIGURE 1:**
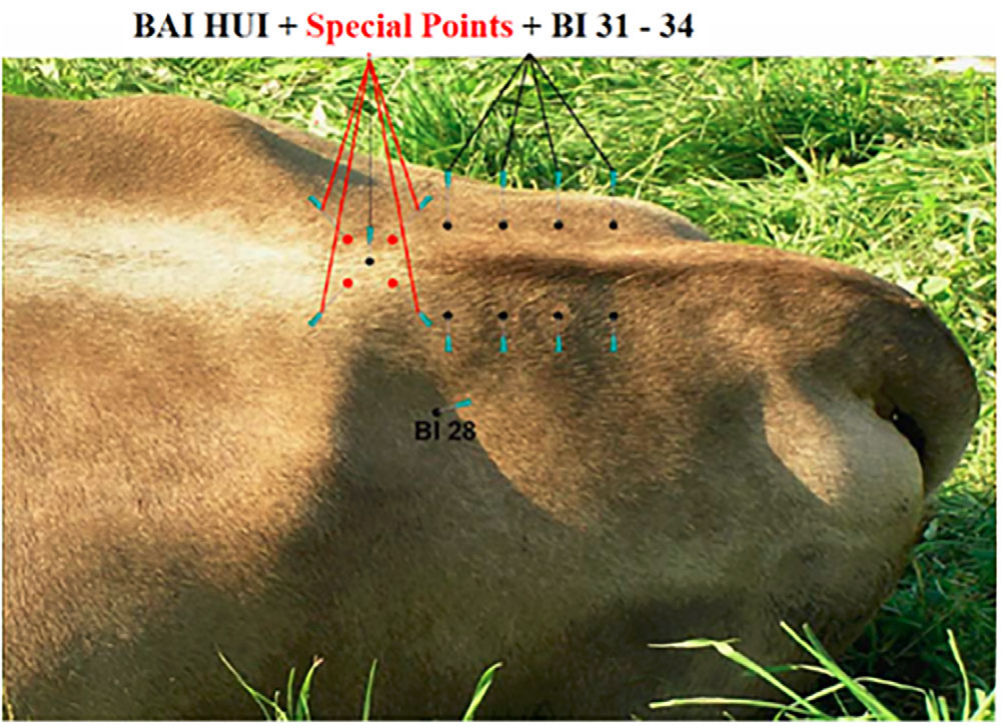
Schematic illustration of the acupuncture points applied in this study to support retorsion and improve insufficient cervical dilatation in cows with intrapartum uterine torsion.

In all animals assigned to Group I, a standardised acupuncture protocol was applied using predefined anatomical landmarks (Figure 1). The following acupoints were consistently treated (Kothbauer and Zerobin 1977; Westermayer 1979; Demmrich‐Wander 2005).

− BAI HUI (point of a hundred meetings): Localisation: Midline point located at the lumbosacral interspace (lumbosacral junction).
− Bladder 31–34 (BL31–BL34): Localisation: Bilaterally over the dorsal sacral foramina, approximately two to three fingerbreadths lateral to the dorsal midline. These points were needled on both sides.
− Bladder 28 (BL28; Back‐Shu point of the urinary bladder): Localisation: Approximately one and a half handbreadths lateral to the dorsal midline, caudal to the iliac crest, at the level of the cranial third of the line between the tuber coxae and the tuber ischiadicum. This point was treated unilaterally.
− Four additional peri‐BAI HUI points (extra points): Localisation: Positioned two to three fingerbreadths lateral to the Bai Hui point and approximately one fingerbreadth cranial or caudal to it. These needles were inserted obliquely in the direction of BAI HUI (Figure 1). All points were identified according to established veterinary acupuncture guidelines and anatomical reference landmarks to ensure reproducibility of the protocol.

### 2.4 Needle Material and Stimulation Duration

Sterile single‐use injection needles were employed for acupuncture treatment (Figure 1). Neoject hypodermic needles (Dispomed, Germany) measuring 0.8 × 40 mm (21G, green) were used in all animals. All acupuncture points were inserted intramuscularly to a depth of approximately 4–5 cm, depending on anatomical location and body condition. The needles were retained in situ until the end of the expulsive phase or until fetal extraction was completed. In cases requiring caesarean section, the needles were removed during surgical preparation.

### 2.5 Assessment of Cervical Canal Passability Before Retorsion

The spatial dimensions and elasticity of the birth canal prior to retorsion were assessed manually by vaginal examination. To ensure intra‐observer consistency, evaluation was standardised using the examiner's hand dimensions as reference values. The hand measured 9 cm in width with the thumb positioned alongside the index finger, 8 cm with the thumb placed against the palm, and 15 cm in maximum span with the fingers fully extended. Based on these reference dimensions and taking tissue elasticity into account, cervical passability was categorised into four groups (< 7 cm, 7–8 cm, 9–13 cm, and 14–18 cm) (Table 1). Even in cases with higher degrees of uterine torsion (180°–360°), complete occlusion of the birth canal was not consistently observed, although cervical dilatation remained insufficient for normal vaginal delivery.

**TABLE 1:** Cervical canal opening prior to retorsion in cows with intrapartum uterine torsion (Group I vs. Group II).

Cervical canal Group	<7 cm *n* (%)	7–8 cm *n* (%)	9–13 cm *n* (%)	14–18 cm *n* (%)	Total *n* (%)
I	2 (4.4)	8 (17.8)	21 (46.7)	14 (31.1)	45 (100)
II	1 (2.1)	6 (12.5)	26 (54.2)	15 (31.3)	48 (100)

*Note*: No statistically significant difference was observed between the two groups (*p* = 0.62).

### 2.6 Retorsion Methods

The time interval between acupuncture needle placement and the initiation of the retorsion procedure was approximately 15 min. The retorsion technique applied in each case was documented. Established correction methods were used as described by Berchtold et al. (1993), including Kamer's maneuver, Caemmerer torsion forceps, and the rolling‐board method. Kamer's maneuver consisted of manual rotation of the fetus using the obstetrician's arm positioned contralateral to the direction of torsion and was feasible only when the birth canal permitted passage of one arm. Mechanical correction using Caemmerer torsion forceps was performed when sufficient cervical passability allowed manual placement of the instrument. The rolling‐board method involved placing the cow in lateral recumbency and rolling her in the direction opposite to the torsion while stabilising the uterus with a weighted plank positioned over the flank region. If adequate cervical dilatation was not achieved after successful retorsion, caesarean section was performed.

### 2.7 Degree of Cervical Dilatation After Retorsion

Post‐retorsion cervical status was evaluated by digital palpation and classified into six categories according to the degree of insufficient cervical dilatation. Complete effacement was defined as the absence of any palpable cervical tissue within the soft birth canal. Grade 1 insufficient dilatation was recorded when the cervical canal was passable for the fetal limbs and head, although the cervix formed a cuff‐like structure around the fetus. Grade 1–2 insufficient dilatation was diagnosed when the canal permitted passage of the limbs and nose, but the cervix extended over the fetal forehead and eyes, thereby preventing passage of the head. Grade 2 insufficient dilatation was defined as a cervical canal passable for either the fetal head or the limbs only. Grade 3 insufficient dilatation indicated that the canal was not passable for fetal parts but allowed passage of the examiner's hand. Grade 4 insufficient dilatation was characterised by a clearly formed but minimally dilated cervix, passable only for a few fingers.

### 2.8 Waiting Time Until Calf Extraction

The time required for further cervical dilatation following retorsion was recorded in minutes. Timing commenced immediately after successful repositioning of the uterus and ended at the initiation of conservative obstetrical extraction, surgical intervention, or the onset of the expulsive phase in spontaneous labour.

### 2.9 Mode of Delivery

Final delivery outcome was categorised as follows: Spontaneous parturition, conservative obstetrical extraction, fetotomy, caesarean section, no obstetrical intervention / euthanasia.

### 2.10 Statistical Analysis

Statistical analyses were performed to compare outcomes between Group I (acupuncture) and Group II (control). The Wilcoxon–Mann–Whitney test was used to evaluate differences in cervical dilatation parameters, degree of insufficient cervical dilatation, and waiting time until calf delivery. Spearman's rank correlation coefficient was calculated to assess the association between the degree of insufficient cervical dilatation and the waiting time to delivery. Statistical analyses were conducted using the software packages Testimate and BMDP/Dynamic Release 7.0. A significance level of *p* < 0.05 was considered statistically significant.

## 3 Results

### 3.1 Opening and Widening of the Cervical Canal Before Retorsion

Prior to retorsion, a slightly higher proportion of cows in the acupuncture group (Group I) exhibited a cervical canal that was impassable or only marginally passable for one hand (≤ 8 cm) compared with the control group (Group II) (22.2% [10/45] vs. 14.6% [7/48], respectively; Table 1). However, no statistically significant difference was detected between Groups I and II regarding cervical canal opening before retorsion (*p* = 0.62; Table 1). The distribution of cervical dilatation grades after retorsion did not differ significantly between groups, indicating comparable baseline conditions with respect to cervical insufficiency. Nevertheless, the time to delivery was significantly shorter in the acupuncture group. This finding suggests that the effect of acupuncture is not solely related to structural cervical changes but may involve functional mechanisms, such as improved myometrial activity or neurophysiological modulation.

### 3.2 Degree of Rotation

No statistically significant difference was observed between Group I and Group II regarding the distribution of uterine torsion degrees (*p* = 0.24). The detailed distribution of torsion severity across both groups is presented in Table 2. In all cases, uterine torsion was classified as incomplete, as at least limited manual access to the fetus or fetal membranes was possible. In addition, torsion occurred predominantly in a left‐sided direction, with 36 cases in Group I and 38 cases in Group II.

**TABLE 2:** Degree of torsion of the uterus in cattle.

Degree of uterine torsion	90° *n* (%)	180° *n* (%)	270° *n* (%)	360° *n* (%)	Total *n* (%)	*p*
Group						
I	1 (2.2)	12 (26.7)	25 (55.6)	7 (15.6)	45 (100)	0.24
II	2 (4.2)	16 (33.3)	26 (54.2)	4 (8.3)	48 (100)	

### 3.3 Retorsion Methods

In Group I (acupuncture), manual retorsion using Kamer's technique was successful in 91.1% of cases (41/45). In two cases, manual retorsion was initially unsuccessful. In one of these animals, torsion forceps according to Caemmerer were subsequently applied, and in one case, due to insufficient cervical passability, the rolling‐board method was performed. In Group II (control), manual retorsion using Kamer's technique was successful in 91.7% of cases (44/48). In three cows, manual correction failed. Of these, one animal was treated using the rolling‐board method, whereas in two cases caesarean section was performed directly without further obstetrical attempts, and in two additional cases laparotomy was performed and subsequently followed by caesarean section. Overall, non‐manual retorsion methods were required less frequently in Group I than in Group II. However, this difference was not statistically significant (*p* = 0.62).

### 3.4 Degree of Cervical Opening After Retorsion

After retorsion, no mechanical dilatation of the cervix was performed, and vaginal delivery was achieved in 41 cows in Group I and 39 cows in Group II. Cervical dilatation was evaluated immediately after successful retorsion in 89 cows (Table 3). Complete cervical effacement was observed in 13.6% of cows in Group I compared with 8.9% in Group II. The distribution of cervical dilatation grades after retorsion did not differ significantly between the two groups (*p* = 0.60).

**TABLE 3:** Degree of cervical insufficiency after conservative retorsion of uterine torsion in cattle (Group I vs. Group II; *n* = 89).

Dilatation of the cervix	Group I *n* (%)	Group II *n* (%)
**Completely dilated**	6 (13.6)	4 (8.9)
**1 degree**	11 (25.0)	18 (40.0)
**1–2 degrees**	18 (40.9)	14 (31.1)
**2 degrees**	5 (11.4)	7 (15.6)
**3 degrees**	3 (6.8)	2 (4.4)
**4 degrees**	1 (2.3)	_
**Total**	44 (100)	45 (100)

*Note*: No statistically significant difference was detected between Group I and Group II in the distribution of post‐retorsion cervical dilatation grades (*p* = 0.60).

### 3.5 Waiting Time Until Calf Extraction

The degree of cervical dilatation after retorsion significantly influenced the interval until calf delivery. Increasing severity of cervical stenosis was associated with a prolonged waiting time. A statistically significant positive correlation was observed between the degree of insufficient cervical dilatation and the time to delivery (Spearman's *r* = 0.59, *p* < 0.0001). No mechanical dilatation of the cervix was performed in either group. Within 10 min after successful retorsion, 72.7% of calves in Group I (acupuncture) were delivered, compared with 44.4% in Group II (control) (Table 4). The mean interval between retorsion and calf delivery was 12.2 ± 1.2 min in Group I and 33.0 ± 1.3 min in Group II, corresponding to an average difference of 20.8 min. Statistical analysis confirmed that the waiting time until delivery was significantly shorter in the acupuncture group than in the control group (*p* = 0.002). The time intervals presented in Table 4 were defined to reflect clinically relevant periods under field conditions rather than a uniform biological progression. Variations in time to delivery may be influenced by factors such as the degree and duration of torsion, cervical tissue characteristics, and fetal size. In Group I, 32 calves (71.1%) were born alive and 13 (28.9%) were stillborn, whereas in Group II, 32 calves (68.1%) were born alive and 15 (31.9%) were stillborn. No statistically significant difference in neonatal outcome was observed between the groups (*p* = 0.89).

**TABLE 4:** Waiting **t**ime to calf delivery (minutes) after successful correction of uterine torsion in Group I (acupuncture) and Group II (control) (*n* = 89).

Waiting time (minutes)	Group I *n* (%)	Group II *n* (%)
≤ 10	32 (72.7)	20 (44.4)
10–59	8 (18.2)	6 (13.3)
60–120	1 (2.3)	10 (22.2)
> 120	3 (6.8)	9 (20.0)
Total	44 (100)	45 (100)

*Note*: A significantly higher proportion of calves were delivered within 10 min after retorsion in Group I compared to Group II (*p* = 0.002).

## 4 Discussion

Obstetrical acupuncture is widely applied in human medicine, particularly for pain management, cervical ripening, and induction or modulation of uterine contractions (Tremeau et al. 1992; Curtis et al. 2006; Ma et al. 2011; Neri et al. 2018). Evidence suggests that acupuncture may facilitate cervical dilatation in women (Hu et al. 2025). In contrast, controlled investigations in veterinary obstetrics remain scarce. To date, no clinical studies have evaluated the effect of acupuncture on cervical dilatation following uterine retorsion in cattle with intrapartum torsio uteri in a larger patient population (Habacher et al. 2006). The present study therefore aimed to determine whether a standardised acupuncture protocol administered prior to retorsion influences cervical dilatation and the subsequent course of parturition in cows. Given that the physiological mechanisms of parturition are fundamentally comparable among cattle, the use of a standardised acupuncture point combination appears justified. The selected protocol was based on previously described obstetrical acupuncture concepts (Westermayer 1979) and later clinical modifications (Weiss and Egel‐Weiss 2006). Rapid delivery following correction of uterine torsion is clinically advantageous, improving both neonatal survival and obstetrical safety. In cases of persistent cervical stenosis after retorsion, prolonged manipulation increases the risk of fetal mortality and may necessitate repeated obstetrical interventions or caesarean section (Erteld et al. 2012; Erteld et al. 2014; Klaus‐Halla et al. 2018; Sickinger et al. 2020). Previous reports have suggested that acupuncture may promote cervical relaxation (Kothbauer and Zerobin 1977; Westermayer 1979); however, the underlying mechanisms remain incompletely understood. Hormonal mediation via relaxin has been hypothesised (Westermayer 1979). Nevertheless, the physiological role of relaxin in cattle remains uncertain, and reliable evidence for its functional significance during bovine parturition is limited (Malone et al. 2017). In contrast, insulin‐like growth factor‐related mechanisms may play a more relevant role in cervical softening in this species (Ivell 1997). Alternatively, segmental neurophysiological mechanisms may contribute to the observed effects. According to segmental regulatory theory, stimulation of somatic afferent fibres may influence autonomic pathways, thereby modulating regional blood flow and organ function (Zohmann 1990). Enhanced cervical perfusion and myometrial activity may thus represent plausible mechanisms. Experimental observations have also indicated that acupuncture may influence uterine contractility, including contraction depth, frequency, and coordination (Kothbauer and Zerobin 1977). Such effects could theoretically reduce the interval between retorsion and delivery, particularly in cases complicated by secondary uterine inertia. In the present study, the observed reduction in time to delivery may be associated with enhanced myometrial activity induced by acupuncture. Neuroendocrine mechanisms are likely involved, including stimulation of the hypothalamic–pituitary axis with subsequent oxytocin release. In addition, activation of the parasympathetic nervous system may promote uterine smooth muscle contraction. Furthermore, acupuncture has been reported to stimulate the synthesis of prostaglandins, such as PGE_2_ and PGF_2_α, which are known to increase the strength and coordination of uterine contractions (Handayani and Balgis 2019; Setiawandari et al. 2023). These combined effects may contribute to more efficient labour progression in cows treated with acupuncture. However, robust controlled studies confirming these findings in cattle are still lacking. Beyond potential effects on cervical tissue and uterine motility, acupuncture is known to activate endogenous analgesic pathways. In both human and animal studies, spinal modulation and the release of endogenous opioids, including endorphins and enkephalins, as well as serotonergic and catecholaminergic mechanisms, have been demonstrated (Pomeranz 1999; Steiss 2003). Reduced stress and improved relaxation of the dam may indirectly support the progression of labour. In addition to its potential effects on uterine contractility, acupuncture may also influence labour progression through analgesic mechanisms. It has been reported that acupuncture stimulates the central nervous system, leading to increased release of neurotransmitters such as β‐endorphin and 5‐hydroxytryptamine (5‐HT) (Qu and Zhou 2007). This neurochemical response not only elevates the pain threshold but may also indirectly promote oxytocin release from the pituitary gland (Nwanodi 2016; Setiawandari et al. 2023). Reduced pain perception and improved relaxation of the dam may therefore contribute to more efficient uterine contractions and coordinated labour activity. However, endocrine or neurochemical parameters were not assessed in the present study, and such mechanisms therefore remain speculative. In the current investigation, the degree of cervical dilatation after retorsion did not differ significantly between groups. Nevertheless, the distribution of severe cervical stenosis was higher in the control group. Surgical intervention was required more frequently in cows that did not receive acupuncture, whereas almost all cows in the acupuncture group delivered vaginally. Although these findings suggest a potential clinical benefit, the sample size was insufficient to demonstrate a statistically significant reduction in caesarean section rates. Importantly, acupuncture significantly reduced the interval between retorsion and adequate cervical opening. Because waiting time increased with the degree of cervical stenosis, this factor was included in a two‐factor covariance analysis. Independent of the initial degree of cervical narrowing, the time to delivery or extraction was significantly shorter in the acupuncture group. From a clinical perspective, shortening this interval may be highly relevant, as prolonged labour is associated with increased maternal and fetal risk. The lack of a significant difference in immediate cervical dilatation, despite a markedly reduced time to delivery, may indicate that acupuncture primarily affects functional aspects of labour, such as uterine contractility and coordination, rather than inducing immediate structural changes in cervical opening. In addition, cervical assessment was performed manually and is therefore subject to a degree of subjectivity; however, this was minimised by the use of a single experienced examiner and standardised evaluation criteria. The study was conducted under field conditions, which inevitably introduced variability. Despite randomisation and defined exclusion criteria, factors such as parity, duration and degree of torsion, fetal–maternal size ratio, and pre‐existing uterine inertia may have influenced outcomes. While this heterogeneity reflects real‐world clinical practice, more standardised experimental conditions could allow clearer mechanistic conclusions. Future investigations should evaluate acupuncture in larger cohorts, include standardised hormonal or physiological measurements, and compare different acupuncture protocols. Additionally, the effect of acupuncture on cervical dilatation in dystocia cases without uterine torsion warrants investigation. In conclusion, the findings of this study indicate that acupuncture may serve as a supportive obstetrical measure in cows with intrapartum uterine torsion, particularly in cases complicated by functional cervical stenosis. Although the precise mechanisms remain unclear, the observed reduction in time to delivery suggests potential clinical and economic relevance. A limitation of the study is the lack of blinding, which may have introduced observer bias. Further controlled studies are required to elucidate the underlying physiological mechanisms and to confirm the reproducibility of these findings. In this context, the absence of a sham acupuncture control group represents a limitation of the present study and should be addressed in future well controlled investigations.

## 5 Conclusion

In the present field study, acupuncture administered prior to manual retorsion of intrapartum uterine torsion did not significantly influence the immediate degree of cervical dilatation following correction. However, a significantly higher proportion of calves were delivered within 10 min after retorsion in Group I compared to Group II (*p* = 0.002). Although the need for surgical intervention was lower in the acupuncture group (with only one case requiring caesarean section), this difference did not reach statistical significance. Nevertheless, the reduction in time to delivery may be of considerable clinical relevance, as prolonged labour following uterine torsion is associated with increased maternal and fetal risk. These findings suggest that acupuncture may serve as a supportive adjunct in the obstetrical management of cows with uterine torsion, particularly in cases characterised by delayed or insufficient cervical dilatation. Although a stratified analysis within individual cervical dilatation grades would be of considerable interest, the limited number of cases within each category did not allow for statistically robust subgroup analyses. Further controlled studies incorporating larger sample sizes and physiological measurements are required to confirm these effects and to elucidate the underlying mechanisms of action.

## Author Contributions

Sait Sendag: conceptualisation, data curation, investigation, methodology, software, validation, visualisation, writing – original draft, writing – review and editing, formal analysis. Eva Maria Erteld: conceptualisation, data curation, investigation, methodology, writing – original draft, writing – review and editing, resources. Axel Wehrend: conceptualisation, data curation, methodology, software, validation, visualisation, investigation, resources, project administration, writing – original draft, writing – review and editing. Mehmet Yildiz: investigation, methodology, writing – original draft, writing – review and editing, supervision.

## Funding

The authors have nothing to report.

## Ethics Statement

Nevertheless, ethical approval for the study protocol was obtained from the Ethics Committee of Justus Liebig University Giessen (Animal Welfare Officer‐Ethics review: 1 December 2025). Informed consent was obtained from all animal owners prior to inclusion in the study. During a 1.5‐year observation period, cases of intrapartum uterine torsion in cattle from several dairy farms in Baden‐Württemberg, Germany, were prospectively enrolled in the study.

## Conflicts of Interest

None of the authors have any conflict of interest to declare.

## Data Availability

The data that support the findings of this study are available on request from the corresponding author.
